# DNA Methylation in Prostate Cancer: Clinical Implications and Potential Applications

**DOI:** 10.1002/cam4.70528

**Published:** 2025-01-09

**Authors:** Romane Muletier, Céline Bourgne, Laurent Guy, Aurore Dougé

**Affiliations:** ^1^ Service d'Oncologie médicale CHU Gabriel Montpied Clermont‐Ferrand France; ^2^ Hématologie Biologique CHU Estaing Clermont‐Ferrand France; ^3^ Équipe d'Accueil 7453 CHELTER Université Clermont Auvergne, CHU Clermont‐Ferrand, Hôpital Estaing Clermont‐Ferrand France; ^4^ Service d'Urologie CHU Gabriel Montpied Clermont‐Ferrand France

**Keywords:** biomarkers, DNA methylation, molecular biology, prostate cancer

## Abstract

**Background:**

Prostate cancer is a common cancer with a variable prognosis. Its management is currently guided by histological and biological markers such as the Gleason score and PSA. Developments in molecular biology are now making it possible to identify new targets for better classification of prostate cancer. Among emerging biomarker, DNA methylation, an epigenetic process, is increasingly being studied in carcinogenesis. Techniques for analyzing DNA methylation are constantly improving, and digital PCR now allows absolute methylation quantification with high sensitivity. These techniques can be performed on circulating tumor DNA.

**Materials & Methods:**

We conducted a literature review of scientific articles addressing the topic of DNA methylation in prostate cancer.

**Results & discussion:**

This review summarizes the different genes whose methylation is involved in carcinogenesis and their clinical implications, both diagnostic and prognostic. Methylation monitoring could also be useful for the prediction of treatment response. However, most studies are retrospective, and prospective studies are needed to validate these data.

## 1 Introduction

Prostate cancer (PC) is the most prevalent cancer in men worldwide, with 1,276,000 new cases reported worldwide in 2018. Despite this high incidence, it ranks as the third leading cause of cancer‐related deaths with 359,000 deaths in 2018 [1]. The median age at diagnosis is 69 years. This disease exhibits varying prognoses, with some patients having indolent cancer while others facing a highly aggressive form. Indeed, 5‐year survival at diagnosis for all stages combined is 93% but drops to 30%–50% at the metastatic stage. Several histological and biological biomarkers have been identified to help in patient management, such as the TNM (Tumor Nodes Metastasis) classification, histological International Society of Urological Pathology (ISUP) Grade and preoperative Prostate Specific Antigen (PSA) level. However, these markers encounter their limitations. For instance, serum PSA levels lack specificity, as they can also become elevated in the presence of urinary infections and benign prostate hypertrophy. Although PSA screening is employed for prostate cancer detection, it may lead to unnecessary biopsies [2]. Notably, repeating the biopsy in some cases could yield positive results in 10%–36% of initially negative biopsies [3]. Therefore, there is a clinical need to identify new biomarkers to guide screening, diagnosis, and therapeutic strategy in PC. Among emerging biomarkers in oncology, DNA methylation was particularly studied during the last few years. DNA methylation is an epigenetic process involved in many functions: gene regulation, genomic imprinting, X chromosome inactivation [4]. In tumoral cell, hypermethylation at the promoter level compacts DNA and prevents transcription factors from binding on the enhancer region. This results in gene expression inhibition and concerns especially genes involved in growth suppression and DNA damage repair [5, 6, 7]. The first clinical applications of DNA methylation analysis were in neuro‐oncology. In glioblastoma, a high‐grade brain tumor, methylation of the *MGMT* (O^6^‐methylguanine‐DNA methyltransferase) promoter is now used as a prognostic and predictive factor for response to temozolomide treatment [8, 9]. Indeed, MGMT encodes a protein involved in DNA repair specifically facilitating the removal of alkyl groups at the O^6^ position of guanine residues. If there is low MGMT activity due to promoter hypermethylation, chemotherapy‐induced lesions remain unrepaired, leading to cell death. In contrast, high MGMT activity will allow to repair these lesions, thereby conferring resistance to alkylating agents [10].

The latest classifications of brain tumors prioritize molecular biology (methylation abnormalities, mutations) rather than histology for tumor classification. These methylation anomalies can be identified both in tumor tissue and more recently in circulating tumor DNA (ctDNA) [11]. In recent years, numerous studies have focused on ctDNA, which presents several advantages. It is a noninvasive method that can be performed with a simple blood test. It also reflects tumor heterogeneity [12]. Various genes and their methylation profiles within prostate cancer have undergone thorough scrutiny. This article provides a comprehensive overview of methylation profiles and their clinical significance.

## 2 Management of Prostate Cancer

The management of localized prostate cancer is based on the D'Amico classification, which considers the histological ISUP Grade (Table 1), preoperative PSA level, and TNM. ISUP Grade is based on Gleason score. The more the Gleason stage is high, the more the prognosis of the disease is unfavorable. The Gleason score has several limitations, mainly because it is based on part of the tumor sample, whereas it may vary within the tumor, potentially leading to underestimation or overestimation of this score. It classifies prostate cancer as low risk, intermediate risk, or high risk of recurrence. Depending on the stage, several treatments may be proposed: active surveillance (only for low‐risk cases), prostatectomy, radiotherapy +/− short (for intermediate‐risk cases), or long (for high‐risk cases) hormone therapy and brachytherapy. The prediction of disease's evolution remains challenging, leading some patients to undergo radical prostatectomy even when the cancer does not significantly threaten to their survival [13]. The monitoring is done through regular PSA testing despite their lack of specificity. Biochemical recurrence (BCR), defined by PSA level > 0,2 ng/mL after radical prostatectomy or by nadir of PSA level + 2 ng/mL after radiotherapy [14, 15], occurs in 20% to 40% of patients after radical prostatectomy and 30% to 50% after external beam radiotherapy [16]. It precedes metastatic progression in 30% of cases [17].

**TABLE 1:** D'Amico classification used for stratifying patients with prostate cancer into risk groups.

Low risk	Intermediate risk		High risk
	Favorable	Unfavorable	
PSA ≤ à 10 ng/mL and ISUP 1 (Gleason 3 ± 3) and T1c or T2a	PSA between 10 and 20 ng/mL		PSA > 20 ng/mL Score ISUP > 3 (Gleason 4 + 4, 4 + 5, 5 + 4, 5 + 5) ≥ T2c
	Score ISUP 2 (Gleason 3 + 4)	Score ISUP 3 (Gleason 4 + 3)	
	T2b		

Patients are also divided into two groups for metastatic disease according to tumor volume. Patients are considered to have a high tumor volume if they have at least one visceral metastasis and/or ≥ 4 bone metastases (including one outside the axial skeleton) [18]. Patients with low tumor volume are eligible for primary radiotherapy followed by treatment with double hormone therapy (1st and 2nd generation) [19]. Patients with high‐volume disease will benefit from triplet therapy combining docetaxel chemotherapy, second‐generation hormone therapy, and androgen suppression (first‐generation hormone therapy) [20]. Median overall survival increased from 3.47 to 5.14 years with the addition of abiraterone in this group of patients.

## 3 Methylation and Cancer

**FIGURE 1:**
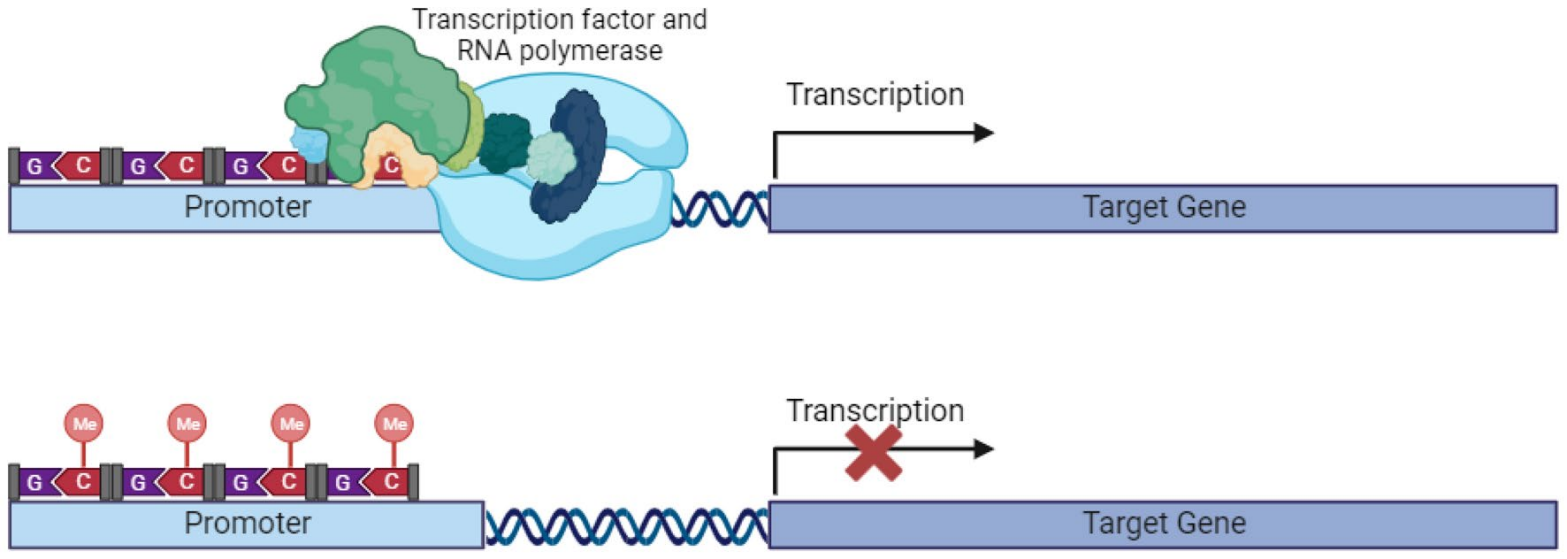
Hypermethylation of the gene promoter prevents the recruitment of transcription factors and RNA polymerase, thereby inhibiting transcription and consequently gene expression.

Methylation is carried out by enzymes called DNA methyl transferases (DNMTs). This is a reversible process involving the covalent addition of a methyl group to the 5′ carbon of a cytosine. These cytosines which are then converted to a 5‐methylcytosine (5mC) are mostly found adjacent to guanine in a complex known as CpG dinucleotides. In mammalian genome, about 80% of CpG dinucleotides are methylated. However, cancer cells present a global hypomethylation and only 40%–60% of CpG dinucleotides are methylated, which disorganizes the chromatin [7], induces genome instability, and could play a role in the activation of oncogenes [21]. CpG dinucleotides can cluster into regions known as ‘CpG islands,’ which are commonly found in gene promoters and are typically unmethylated [22]. Methylation of these sites prevents the recruitment of transcription factors (Figure 1), leading to inhibition of gene expression and playing a role in carcinogenesis [5, 6, 7]. Indeed, when CpG sites within promoters are methylated, they attract proteins such as methyl‐CpG‐binding domain proteins (MBDs), which in turn recruit repressor complexes containing histone deacetylases (HDACs) and histone methyltransferases (HMTs). HDACs remove acetyl groups from histone tails, leading to chromatin condensation and reduced gene accessibility. Simultaneously, HMTs add methyl groups to histone residues, promoting a repressive chromatin state. This combination of DNA methylation and histone modification reinforces transcriptional repression, effectively silencing the gene [23].

Methylation patterns evolve with age and appear to reflect biological aging [24, 25]. Methylation changes in cancer can arise in the initial cell that was transformed by the effects of aging. Mutations in key methylation proteins such as Ten Eleven Translocation (TET) proteins and DNMTs are found in various cancers. TET proteins are involved in DNA demethylation [26], and their dysfunction leads to hypermethylation whereas DNMTs are involved in DNA methylation. These abnormalities are common in hematopoietic malignancies and account for up to 26% of myelodysplastic syndromes (MDS) [27]. They are less common in solid tumors, with a frequency of up to 10% in some studies [26]. In addition, loss of TET protein expression can be caused by abnormalities in other genes, such as *Isocitrate Dehydrogenase 1* (*IDH1*) in glioblastoma, which also leads to hypermethylation of CpG islands [4, 25, 28]. Aberrant methylation by DNMTs leads to the hypermethylation of tumor suppressor genes such as BRCA1 involved in the development of breast cancer [29]. DNMTs are used as therapeutic targets. Indeed, their inhibition allows for the reactivation of tumor suppressor genes expression. In general, methylation changes, particularly hypomethylation, can lead to genomic instability conducive to the development of cancer cells [23]. For example, 5‐azacytidine is used in clinical practice to treat patients with hematological disorders like MDS. Several clinical trials are underway for solid tumors, either with other drugs alone or in combination with immunotherapy [30, 31].

Moreover, studying methylation could help identify the primary origin of carcinoma of unknown origin [32, 33]. Methylation research already has clinical applications. In colorectal and endometrial cancer, hypermethylation of the *MutL homolog 1* (*MLH1*) gene promoter in tumor tissue is part of the rationale for the management of tumors with microsatellite instability. The presence of hypermethylation of the *MLH1* promoter indicates a somatic abnormality of microsatellite instability and therefore rules out the presence of constitutional Lynch syndrome [34, 35]. In ovarian cancer, tumors with a Homologous Recombination Defect (HRD) are eligible for maintenance treatment with PARP inhibitors. Half of these HRD tumors show hypermethylation of the *BRCA1* promoter [36]. As methylation analysis is faster and less costly than HRD status assessment, this intermediate step could reduce the number of HRD status tests in these cancers. Methylation analysis of *SHOX2* and *PTGER4* genes in lung cancer led to the launch of Epi ProLung in the United States. This in vitro diagnostic test is based on ctDNA and can be used to screen for lung cancer [37]. For colorectal cancer, two methylation‐based tests are on the market. The Epi ProColon 2.0 screening test, approved by the FDA in 2017, is based on hypermethylation of the *SEPT9* gene [37]. Initial results were very encouraging, with a sensitivity of 75% and specificity of around 90%. However, the prospective multicentre PRESEPT study showed disappointing results, with a sensitivity of 48% and specificity of 92% [38, 39]. The second test, COLVERA, is based on the methylation of *IKFZ1* and *BCAT1*. It can be used to diagnose recurrence after surgery with a sensitivity of 68% and specificity of 87%. In contrast, the sensitivity of CarcinoEmbryonic Antigen (CEA, a biomarker used in current practice) in this situation is only 32% with a specificity of 94% [40].

In clinical practice, studying methylation is facilitated by liquid biopsies, where circulating tumor DNA (ctDNA) can be analyzed. Analysis of ctDNA is easy to implement in clinical routine because it is accessible through a non‐invasive sampling method [11]. It also reflects tumor heterogeneity unlike targeted biopsy [12]. However, it circulates in low quantities, so working with sensitive techniques is necessary, such as droplet digital PCR (ddPCR).

## 4 Application of Methylation in Prostate Cancers

Methylation can be used to aid in screening and diagnosis (Table 2). Several studies have also examined its correlation with already known biomarkers like PSA, Gleason score or TNM (Table 3). Finally, several studies have focused on its impact on biochemical recurrence, progression‐free survival, and overall survival (Table 4). Here, we describe key gene whose promoter methylation has been investigated for their potential utility in prostate cancer.

**TABLE 2:** Main genes whose promoter hypermethylation is implicated in prostate adenocarcinomas at diagnosis.

Genes	Role	Method	Material	Disease stage	Cases/Controls	Sensitivity	Specificity	Reference
*AOX1*	Production of hydrogen peroxyde	Quantitative Methylation Specific PCR	Prostate tumor tissue	Localized	293/33	71%	97%	[41]
*APC*	Tumor Suppressor Gene	Quantitative Methylation Specific PCR	Prostate tumor tissue	Localized	73/25	90.4%	96.0%	[42]
		Methylation Specific PCR	Prostate tumor tissue	Localized	170/69	64.1%	91.3%	[43]
		Quantitative Methylation Specific PCR	Plasma samples	Localized	42/22	92.9%	91.0%	[44]
*CDH13*	Adhesion cell	Methylation Specific PCR	Plasma samples	No precision	98/47	44.9%	100%	[45]
*EPHA7*	Signal transduction	Quantitative Methylation Specific PCR	Prostate tumor tissue	Localized	48/36	41.70%	83.33%	[46]
*ERα*	Estrogen receptor	Quantitative Methylation Specific PCR	Plasma samples	Localized	84/37	9.50%	100%	[47]
*ERβ*	Estrogen receptor	Quantitative Methylation Specific PCR	Plasma samples	Localized	84/37	20.20%	100%	[47]
*GAS6*	Cell proliferation	Quantitative Methylation Specific PCR	Prostate tumor tissue	Localized	293/33	93%	71%	[41]
*GSTP1*	Intracellular detoxification/DNA damage repair	Methylation Specific PCR	Prostate tumor tissue	Localized	69/31	91.3%	70.9%	[48]
			Plasma samples			36.2%	100%	
		Restriction endonuclease quantitative PCR	Plasma samples	Localized and Metastatic	103/35	12.0%	100%	[49]
		Methylation Specific PCR	Prostate tumor tissue	Localized and Metastatic	17/15	94.0%	100%	[13]
			Plasma samples	Localized and Metastatic	32/22	72.0%	100%	
		Quantitative Methylation Specific PCR	Plasma samples	Localized	42/22	95.0%	91.0%	[44]
		Quantitative Methylation Specific PCR	Prostate tumor tissue	Localized	73/25	93.2%	100.0%	[42]
*HAPLN3*	Adhesion cell	Quantitative Methylation Specific PCR	Prostate tumor tissue	Localized	293/33	71.0%	97%	[41]
		Droplet Digital PCR	ctDNA	Localized	27/10	44.0%	100%	[50]
*MCAM*	Adhesion cell	Quantitative Methylation Specific PCR	Plasma samples	Localized	84/37	65.50%	73.30%	[47]
*MDR1*	Molecules transporters intra and extra‐cellular Involve in drug resistance	Quantitative Methylation Specific PCR	Prostate tumor tissue	Localized	73/25	87.70%	92%	[42]
*PITX2*	Transcriptional regulation	Quantitative Methylation Specific PCR	Prostate tumor tissue	Localized	25/50	80%	96%	[51]
*PTGS2*	Inflammatory response	Quantitative Methylation Specific PCR	Prostate tumor tissue	Localized	73/25	87.70%	100%	[42]
*RARB2*	Growth cell regulation	Quantitative Methylation Specific PCR	Prostate tumor tissue	Localized	118/68	94.9%	100.0%	[52]
		Quantitative Methylation Specific PCR	Plasma samples	Localized	42/22	78.6%	91.0%	[44]
*RASSF1A*	Tumor Suppressor Gene	Quantitative Methylation Specific PCR	Prostate tumor tissue	Localized	73/25	95.9%	100.0%	[42]
		Quantitative Methylation Specific PCR	Plasma samples	Localized	42/22	97,6%	77.0%	[44]
*ST6GALNAC3*	Adhesion cell	Droplet Digital PCR	ctDNA	Localized	27/10	31.0%	100%	[50]
		Quantitative Methylation Specific PCR	Prostate tumor tissue		169/37	70.4%	100%	
*TIMP‐2*	Tumor Suppressor Gene	Methylation Specific PCR	Prostate tumor tissue	Localized	42/32	78.50%	84.30%	[53]
*ZNF660*	Transcriptional regulation	Quantitative Methylation Specific PCR	Prostate tumor tissue	Localized	169/37	68.6%	100%	[50]
		Droplet Digital PCR	ctDNA		27/10	22.0%	100%	

**TABLE 3:** Correlation between prognostic factors and hypermethylation in prostate cancer.

Genes	Role	Method	Material	Disease stage	Cases	Impact	*p*	Reference
*APC*	Tumor Suppressor Gene	Quantitative Methylation Specific PCR	Prostate tumor tissue	Localized	118	Correlation with Gleason Correlation with T stage	*p* = 0.02 *p* = 0.002	[54]
		Quantitative Methylation Specific PCR	Prostate tumor tissue	Localized	25	Correlation with Gleason	*p* = 0.021	[51]
		Pyrosequencing	Prostate tumor tissue	Localized	24	Correlation with Gleason	*p* = 0.02	[55]
*CDH13*	Adhesion cell	Quantitative Methylation Specific PCR	Prostate tumor tissue	Localized	101	Correlation with Gleason Correlation with PSA	*p* < 0.0001 *p* = 0.0003	[56]
		Methylation Specific PCR	Plasma Samples	No precision	98	Correlation with Gleason Correlation with T stage Correlation with PSA	*p* = 0.0395 *p* = 0.0001 *p* = 0.0071	[45]
*CDKN4A*	Cell Cycle Control Tumor Suppressor Gene	Pyrosequencing	Prostate tumor tissue	Localized	24	Correlation with Gleason	*p* = 0.04	[55]
*E‐Cadherin*	Adhesion cell	Quantitative Methylation Specific PCR	Prostate tumor tissue	Localized	82	Correlation with Gleason Correlation with T stage	*p* < 0.05 *p* < 0.05	[57]
*EDNRB*	Angiogenesis regulation	Quantitative Methylation Specific PCR	Prostate tumor tissue	Localized	73	Correlation with Gleason Correlation with T Stage	*p* = 0.005 *p* = 0.04	[42]
*EPHA7*	Signal transduction	Quantitative Methylation Specific PCR	Prostate tumor tissue	Localized	20	Correlation with Gleason	*p* = 0.04	[46]
*GSTP1*	Intracellular detoxification/DNA damage repair	Methylation Specific PCR	Plasma Samples	HRPC	62	Correlation with Lymph Node metastases	*p* = 0.02	[58]
		Pyrosequencing	Prostate tumor tissue	Localized	24	Correlation with Gleason	*p* = 0.01	[55]
		Quantitative Methylation Specific PCR	Prostate tumor tissue	Localized	101	Correlation with Gleason Correlation with PSA	*p* < 0.0001 *p* = 0.0003	[56]
		Quantitative Methylation Specific PCR	Prostate tumor tissue	Localized	25	Correlation with Gleason Correlation with T stage	*p* = 0.021 *p* = 0.06	[51]
*GSTP1*		Quantitative Methylation Specific PCR	Prostate tumor tissue	Localized	118	Correlation with Gleason Correlation with T stage	*p* = 0.019 *p* = 0.00004	[54]
*HAPLN3*	Adhesion cell	Droplet Digital PCR	ctDNA	Localized	27	Correlation with T stage	*p* = 0.032	[50]
*HOXD3*	Transcriptional regulation	Quantitative Methylation Specific PCR	Prostate tumor tissue	Localized	66	Correlation with Gleason	*p* = 0.014	[59]
*RARB2*	Growth cell regulation	Quantitative Methylation Specific PCR	Prostate tumor tissue	Localized	101	Correlation with Gleason	*p* < 0.0001	[56]
		Quantitative Methylation Specific PCR	Prostate tumor tissue	Localized	25	Correlation with Gleason	*p* = 0.018	[51]
		Quantitative Methylation Specific PCR	Prostate tumor tissue	Localized	118	Correlation with T stage	*p* = 0.0009	[52]
*RASS1FA*	Tumor Suppressor Gene	Quantitative Methylation Specific PCR	Prostate tumor tissue	Localized	118	Correlation with T stage	*p* = 0.0025	[54]
		Quantitative Methylation Specific PCR	Prostate tumor tissue	Localized	101	Correlation with Gleason Correlation with PSA	*p* < 0.0001 *p* = 0.0003	[56]
*SDR5A2*	Androgen metabolism	Pyrosequencing	Prostate tumor tissue	Localized	86	Correlation with PSA	*p* = 0.01	[60]
*ZNF660*	Transcriptional regulation	Droplet Digital PCR	ctDNA	Localized	27	Correlation with T stage	*p* = 0.006	[50]
		Quantitative Methylation Specific PCR	Prostate tumor Tissue	Localized	169	Correlation with Gleason	*p* = 0.0077	[50]

**TABLE 4:** Impact of hypermethylation on prostate cancer prognosis.

Genes	Role	Method	Material	Disease stage	Cases	Impact	*p*	Reference
*APC*	Tumor Suppressor Gene	Methylation Specific PCR	Prostate Tumor Tissue	No precision	280	Prognosis: specific prostate cancer mortality	*p* = 0.02	[61]
		Quantitative Methylation Specific PCR	Prostate Tumor Tissue	Localized Gleason 7	74	Prognosis: biochemical recurrence	*p* = 0.004	[62]
*C1orf114*	Pseudogene, no function known	Quantitative Methylation Specific PCR	Prostate Tumor Tissue	Localized	114	Prognosis: biochemical recurrence	*p* = 0.0268	[41]
*CDH13*	Cell adhesion	Methylation Specific PCR	Prostate Tumor Tissue	Localized	151	Prognosis: biochemical recurrence	*p* = 0.02	[63]
			Plasma samples	No precision	98	Prognosis: overall survival	*p* = 0.0073	[45]
*CDKN2A*	Tumor Suppressor Gene	Methylation Specific PCR	Prostate Tumor Tissue	Localized	151	Prognosis: biochemical recurrence diminution	*p* = 0.05	[63]
*CYP11A1*	Steroid metabolism	Pyrosequencing	Prostate Tumor Tissue	Localized	189	Prognosis: biochemical recurrence	*p* < 0.0001	[60]
*DOCK2*	Immune response	Quantitative Methylation Specific PCR	Prostate Tumor Tissue	Localized	234	Prognosis: biochemical recurrence	*p* = 0.001	[62]
						Prognosis: time to recurrence	*p* = 0.016	
*GRASP*	Intracellular organization	Quantitative Methylation Specific PCR	Prostate Tumor Tissue	Localized	234	Prognosis: biochemical recurrence	*p* = 0.003	[62]
						Prognosis: time to recurrence	*p* = 0.019	
*GSTP1*	Intracellular detoxification/DNA damage repair	Quantitative Methylation Specific PCR	Prostate Tumor Tissue	Localized Gleason 7	74	Prognosis: biochemical recurrence	*p* = 0.004	[62]
		Restriction endonuclease quantitative PCR	Plasma samples	Localized	55	Prognosis: biochemical recurrence	*p* < 0.001	[49]
*HIF3A*	Transcription factor	Quantitative Methylation Specific PCR	Prostate Tumor Tissue	Localized	234	Prognosis: biochemical recurrence	*p* = 0.024	[62]
						Prognosis: time to recurrence	*p* = 0.037	
*PFKP*	Glycosylis Regulation	Quantitative Methylation Specific PCR	Prostate Tumor Tissue	Localized	234	Prognosis: biochemical recurrence	*p* = 0.006	[62]
						Prognosis: time to recurrence	*p* = 0.037	
*PTGS2*	Inflammatory response	Quantitative Methylation Specific PCR	Prostate Tumor Tissue	Localized	36	Prognosis: biochemical recurrence	*p* = 0.0017	[42]
*ZNF660*	Transcriptional regulation	Quantitative Methylation Specific PCR	Prostate Tumor Tissue	Localized	169	Prognosis: biochemical recurrence	*p* = 0.004	[50]

### 4.1 Glutathione S‐Transferase Pi 1 (GSTP1)

*GSTP1* encodes an enzyme involved in cellular detoxification with antioxidant function, thereby playing a protective role on DNA [64]. *GSTP1* methylation has been studied since 1994 and is the most frequently alteration observed in prostate cancer [65]. In prostate adenocarcinoma tissue samples, hypermethylation of the *GSTP1* promoter occurred in more than 90% of cases [48]. Its specificity ranged from 70% to 100%, with a sensitivity of 91.3% to 94% [42, 66]. In ctDNA from plasma, its specificity was high (90%–100%), but its sensitivity was lower (15%–72%) [44, 48, 49, 58, 66]. Methylation patterns of *GSTP1* in tumor tissues displayed a significant association with the Gleason stage [54, 55]. Similar findings were observed with preoperative PSA levels, where the methylation index showed a significant correlation with elevated PSA levels (*p* = 0.0003) [56]. A parallel correlation was established between hypermethylation of *GSTP1* and tumor stage (*p* = 0.00004) [63]. In serum samples of patients with hormone‐resistant prostate cancer, hypermethylation of *GSTP1* was also linked to lymph node metastases (*p* = 0.02) [58]. The hypermethylation of *GSTP1* detected in preoperative serum plasma was associated with BCR (HR 4.4; 95% CI 2.2, 8.8; *p* < 0.001) after multivariable analysis [49]. Instead, methylation of *GSTP1* in tumor tissues showed no correlation with biochemical recurrence or prostate cancer mortality [61, 63], except when focusing on Gleason 7 (3 + 4) PC (*p* = 0.004) [62]. Rouprêt et al. showed that the level of methylation *of GSTP1* in ctDNA from serum plasma increased at the time of BCR [44]. To our knowledge, only one study was published about the correlation between methylation gene profile and the therapeutic response of PC. In this study, *GSTP1* methylation was analyzed in 562 patients with metastatic castration‐resistant prostate cancer treated with docetaxel. *GSTP1* methylation was found in 458 patients. It correlated with worse overall survival (OS) independently of other risk factors (*p* < 0.00001). OS was about 20 months for patients with *GSTP1* methylation, whereas OS exceeded 30 months for patients without *GSTP1* methylation. They then analyzed the evolution of methylation in patients after 2 cycles of chemotherapy. Undetectable level was observed in 243 (53%) patients and was associated with a better OS (24 months vs. 14 months if detectable methylation; *p* < 0.00001) [67].

### 4.2 Adenomatous Polyposis Coli (APC)

*APC* belongs to the tumor suppressor gene family and its protein plays a role in cell invasion. The *APC* promoter was shown to be hypermethylated in prostate tumors with a sensitivity of 64%–90% and a specificity of 91%–96% [42, 43]. In a study analyzing the methylation patterns of 10 genes in the plasma of 42 patients with prostate cancer and 22 control patients, a significant hypermethylation difference was observed for four genes, including *APC* (*p* < 0.0001) [44].

Regarding the correlation with biomarkers, hypermethylation of *APC* in tumor tissue was correlated with Gleason stage and TNM pathological stage (*p* = 0.002) [54, 55]. In the population of Gleason 7 (3 + 4) PC, hypermethylation of *APC* was also correlated with BCR (*p* = 0.004) [62]. It is noteworthy to concentrate on the Gleason 7 population because this is the most heterogeneous group of patients, providing valuable insights for discovering new biomarkers. In addition to being associated with BCR, hypermethylation of *APC* was also correlated with prostate cancer mortality (HR = 1.57; 95% CI, 0.95–2.62) [61].

### 4.3 Retinoic Acid Receptor Beta 2 (RARβ2)

The methylation status of the RARβ2 promoter has been studied in various cancers, including lung, breast, and colorectal cancers [68, 69, 70]. It encodes a nuclear transcription factor involved in the regulation of cell growth. Hypermethylation of *RARβ2* was present in prostate cancer but also in high‐grade prostatic intraepithelial neoplasia (HGPIN) and BPH, though in lower quantities. Jeronimo et al. demonstrated, by setting the level (RARß2/β‐actin, reference gene) to 1, that it is possible to achieve differentiation between cancerous and non‐cancerous tissue with a sensitivity of 94.9% and a specificity of 100% [52]. With this cut‐off, the median methylation levels were 0, 87.6, and 234.7, respectively, for BPH, HGPIN, and PC. Maryuma et al. demonstrated that methylation index of *RARβ2* was significantly higher in high Gleason‐grade tumors (*p* < 0.0001) [56]. Methylation levels of *RARβ2* were additionally associated with pathological tumor stage (*p* = 0.0009) [52]. No study has demonstrated its correlation with BCR but level of methylation of *RARβ2* increased at the time of biochemical recurrence [44].

### 4.4 Cadherin 13 (CDH13)

This gene encodes Cadherin‐13, a protein involved in cell adhesion. *CDH13* was hypermethylated in ctDNA from plasma of patients with prostate cancer (44.9%), while no methylation was found in control patients (*p* < 0.0001) [45]. Furthermore, hypermethylation of *CDH13* in plasma correlated with tumor stage (*p* = 0.0001) and PSA levels (*p* = 0.0071) [45]. Hypermethylation in tumor was also associated with Gleason score (*p* = 0.0395) and PSA levels [45, 56]. In an analysis of 15 gene methylation profiles on prostate tumoral tissue of 151 patients, *CDH13* was the single gene associated with biochemical recurrence after adjusting for other factors (*p* = 0,02) [63]. These results were confirmed in overall survival in a cohort of 98 patients after adjusting for risk factors, with a relative risk of death of 6.132 (95% CI 3160–12,187, *p* = 0,0073) [45].

#### 4.4.1 Other Genes

Many other genes have been studied. In addition to *GSTP1* and APC, Yegnasubramian et al. demonstrated that hypermethylation of *RASSF1a, MDR1*, and *PTGS2* had a sensitivity of 95.9%, 87.7%, and 87.7%, respectively, with specificities of 100%, 92%, and 100%, respectively [42]. Haldrup et al. showed that *ST6GALNAC3* and *ZNF660* were significantly hypermethylated in localized PC compared to other prostate samples (normal adjacent tissue, benign prostatic hyperplasia (BPH), prostatic intraepithelial neoplasia (PIN)) [50]. Hypermethylation of *ZNF660* (*p* = 0.0077), *EDNRB* (*p* = 0.005), *EphA7* (*p* = 0.04), and *E‐Cadherin* (*p* < 0.05) was correlated with Gleason stage [42, 45, 46, 50, 51, 57]. Similarly, a parallel correlation was established between hypermethylation of *RASSF1A*, *EDNRB*, and *E‐Cadherin* in prostate tumors and the TNM pathological stage (*p* = 0.0025, *p* = 0.04, and *p* < 0.05, respectively) [42, 54, 57]. Conversely, hypermethylation of *HOXD3* was not an effective biomarker for prostate cancer detection (Se = 16.6%) but was correlated with the Gleason score (*p* = 0.014). Its methylation level also correlated with PSA levels (*p* = 0.03) [59].

Cottrell et al. conducted an initial analysis of genome methylation (methylome). Following this initial step, they selected 62 candidate genes. Among these genes, *GPR7*, *ABHD9*, and *Chr3‐EST*, were significantly hypermethylated in the BCR group compared to the no BCR group. They confirmed these results in Methylation Real‐Time PCR for *ABHD9* and *Chr3‐EST* in 223 patients. The effect was consistent in a multivariate logistic analysis for predictive markers (Gleason score, pathological stage, surgical margin status) [71]. *ZNF660* hypermethylation in tumor tissue was also correlated with BCR (*p* = 0,004), but this association was not confirmed after adjusting for routine predictor factors (*p* = 0,092) [50]. Furthermore, the methylation level for *ZNF660* was significantly correlated with the disease stage: localized PC versus metastatic PC (*p* = 0.002) [50]. In a small cohort of 36 patients, a high level of methylation of *PTGS2* was correlated with the risk of BCR (*p* = 0,0017) [42]. Hypermethylation of *CYP11A1* in primary prostate tumors was also associated with biochemical recurrence (*p* < 0.0001), with similar results on plasma samples [60]. On the contrary, methylation of *CDKN2A* was correlated with a decreased risk of biochemical recurrence (OR 0,43; 95% CI 0.19–0,98; *p* = 0,05) [63].

#### 4.4.2 Panel of Genes

For more complete analyses, panels with multiple genes have been developed. As an example, Enodika et al. showed that the combination of methylation of *GSTP1*, *APC*, and *MDR1* analyses led to a sensitivity of 65.4% and specificity of 94.2% [43]. The combination of *ERα*, *ERβ*, and *MCAM* in serum increased sensitivity to 75% and specificity to 70% [47]. Van Neste et al. developed Episcore, which consists of the detection of methylation levels of *GSTP1*, *APC*, and *RASSF1* promoter genes in prostate‐negative biopsies. The absence of methylation exhibits a negative predictive value of 96% for high‐grade cancer and reduces the number of unnecessary biopsies by 3.3 to 5 times [72]. In a panel of three genes, methylation of *GSTP1*, *APC*, and *MDR1* in prostate tumors was significantly associated with pathological stage (*p* < 0.001), Gleason score (*p* < 0.001), capsular involvement (*p* < 0.001), vesicle invasion (*p* = 0.002), and pelvic lymph node metastasis (*p* = 0.001) [43]. From a prognostic perspective, the association of hypermethylation of *AOX1*, *C1orf114*, and *HAPLN3* was correlated with biochemical recurrence [41].

The study of DNA methylation could thus serve as a valuable diagnostic tool for prostate cancer. Its analysis in negative prostate biopsies may guide urologists in deciding whether repeat biopsies are necessary. Additionally, its detection in circulating tumor DNA could complement PSA as a screening aid. DNA methylation studies may also refine existing biomarkers. Monitoring methylation in circulating tumor DNA could facilitate the early detection of biochemical recurrences. Furthermore, it holds potential as a marker for therapeutic response. In vitro studies have demonstrated that prostate cancer cell lines exhibit distinct methylation profiles based on their androgen sensitivity [73]. This observation suggests that a tumor's methylation profile may influence its responsiveness to hormone therapy. Moreover, these profiles may evolve during disease progression in response to therapeutic interventions, potentially contributing to the emergence of castration resistance. However, to date, no study has systematically examined the correlation between methylation profiles and response to hormone therapy.

## 5 Methylation Quantification Methods

**FIGURE 2:**
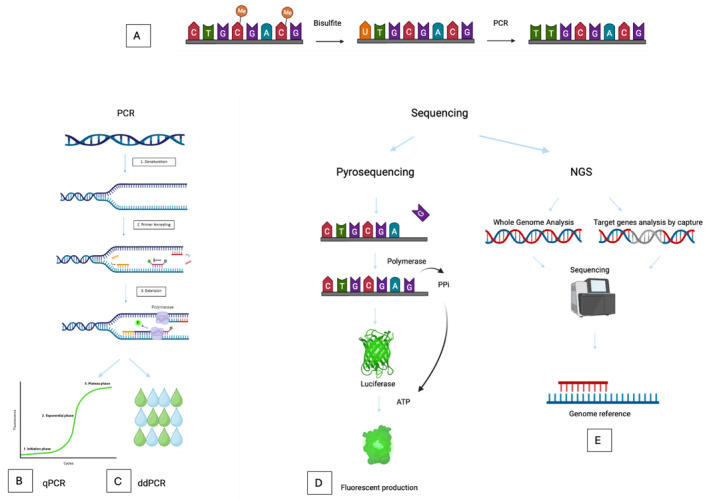
Main techniques for methylation analysis. (A) Bisulfite treatment converts unmethylated cytosine in uracil and differentiates between methylated and unmethylated cytosines. (B) Quantitative PCR (qPCR) allows relative quantification based on a ratio to a reference gene. (C) By generating droplets, droplet digital PCR (ddPCR) provides absolute quantification with improved sensitivity. (D) In pyrosequencing, fluorescence is produced by luciferase activated by ATP released when the base is incorporated by polymerase. (E) In contrast to other techniques, NGS is able to provide a global analysis of the methylome.

Different methods are currently available for DNA methylation analysis (Figure 2). Most of them employ bisulfite treatment to differentiate methylated from unmethylated cytosines. Bisulfite induces a conversion of unmethylated cytosine to uracil, a reaction that remains unfeasible with methylated cytosine [74]. Currently, bisulfite treatment stands as the gold standard technique for quantifying DNA methylation.

### 5.1 Methylation‐Specific Polymerase Chain Reaction (PCR)

*Quantitative Methylation‐Specific PCR* (*qMSP*) is a relative quantification technique based on PCR and using primers specific to methylated or unmethylated DNA after bisulfite conversion. A reference gene insensitive to CpG methylation status allow to normalize results. It can also be used to analyze multiple targets by multiplexing [75]. It is currently the preferred technique used to study methylation in specific genes or loci in oncology, mainly because of its speed and cost‐effectiveness.

*Digital PCR* (*dPCR*) is the “3rd generation PCR technique”. The first step is to divide the total reaction volume of a conventional PCR reaction into a large number of tiny volume compartments in the form of droplets according to a statistical distribution based on Poisson's law [76]. The PCR product is detected at the endpoint using fluorescence detection. Each compartment is considered positive (presence of amplification) or negative (absence of amplification). Like qMSP this technology is based on the use of probes specific to methylated or unmethylated DNA after bisulfite conversion. This detection provides absolute quantification. This strategy is highly sensitive and well‐suited to detect rare events. It is perfectly suited for analyses of ctDNA. Combining fluorochromes can also analyze multiple targets in a single reaction (up to 15 genes of interest) [77]. Some methylation studies begin to appear with this new technique on solid tumors or ctDNA [50, 78, 79]. These techniques are easy to use in a clinical setting.

### 5.2 Methylation Arrays

Methylation arrays are high‐throughput technologies used to assess DNA methylation patterns across the genome. These arrays enable researchers to measure methylation status at thousands of CpG sites simultaneously [80]. Prior to hybridization, DNA must undergo bisulfite conversion. In the most commonly used platforms, two types of probes are designed to target each CpG site: one probe hybridizes with the methylated sequence, while the other targets the unmethylated sequence. Fluorescent signals from these probes are measured, and the ratio between methylated and unmethylated signals provides a quantitative readout. This technique provides high‐throughput, cost‐effective analysis of large samples for extensive methylome analysis at reasonable cost, but is limited to pre‐selected loci.

### 5.3 Sequencing Technologies

Methylation can also be analyzed using *Next Generation Sequencing (NGS)* enabling high‐throughput, genome‐wide analysis with high resolution. NGS‐based methylation studies typically rely on bisulfite conversion, or more recently enzymatic conversion, limiting the degradation associated with bisulfite treatment. This technique can be used to analyze methylation on the whole genome (whole genome bisulfite sequencing (WGBS)) or target sequences using enrichment methods, such as methylated DNA immunoprecipitation sequencing (MeDIP‐Seq) which uses antibodies against 5‐methylcytosine to isolate methylated fragments before sequencing. The sequencing data is then assembled and aligned to a reference genome [81]. Bioinformatics software can be used to analyze methylation sites on a genomic scale by prior conversion to bisulfite. New direct sequencing methods, such as nanopore sequencing, avoid the need for chemical conversion by detecting methylation directly during sequencing. These methods offer long read capabilities, enabling analysis of methylation in repetitive genomic regions. These techniques are mainly used for research, they are challenging to implement in routine clinical practice due to their cost and time requirements.

## 6 Discussion/Conclusion

Methylation data in prostate adenocarcinomas are promising as diagnostic aids and prognostic factors. Identifying new biomarkers in metastatic prostate adenocarcinoma could allow personalized treatments according to patient methylation status. This could have an impact on several key points in their management. Firstly, at the start of 1st‐line metastatic treatment, identifying an unfavorable methylation status could lead to an intensification of the treatment. However, a favorable methylation status in a high‐volume patient who may not tolerate the association of hormonal therapy and chemotherapy (older patient, comorbidities) could help oncologists to de‐escalate. Secondly, the absence or presence of a decrease in methylation during treatment could be an argument to change the line of treatment, particularly in the case of dissociated responses (biological progression and radiological stability). Finally, the identification of targets suggesting a better response to chemotherapy than to hormone therapy could guide the choice of subsequent lines of treatment. Prospective studies are imperative to validate these hypotheses and assess their clinical applicability. Moreover, methylation aberrations may potentially serve as therapeutic targets. For instance, 5‐azacytidine, used as a treatment for myelodysplastic syndromes and acute myeloid leukemia, acts as a hypomethylating agent. In vitro studies have demonstrated its ability to restore the expression of *TIMP‐2*, previously suppressed by promoter hypermethylation in PC cell lines [53]. Over the past two decades, the progress of molecular biology led to the identification of novel biomarkers. As a result, patient management in oncology is evolving toward increasingly personalized medicine. Epigenetic data constitute a significant component of these emerging targets and have already played a pivotal role in the management of glioblastomas. Extensive studies have investigated the hypermethylation of numerous genes in retrospective analyses of prostate adenocarcinomas. Most of these studies employed quantitative methylation‐specific PCR, enabling only relative quantification of methylation levels. Advancements in techniques such as digital PCR enhanced the analysis of these data by increasing sensitivity, thereby facilitating the detection of rare events. This methodology is well‐suited for analyses involving circulating tumor DNA and provides absolute quantification. Like qPCR, dPCR can be integrated into routine clinical practice at a reasonable cost. In third‐generation sequencing techniques, long‐read sequencing also enables comprehensive genome‐wide methylation analysis. Currently, these techniques are primarily confined to clinical research applications but will likely unveil new biomarkers or novel combinations shortly [82, 83].

## Author Contributions

**Romane Muletier:** conceptualization (lead), writing – original draft (lead), writing – review and editing (lead). **Céline Bourgne:** supervision (equal), writing – review and editing (equal). **Laurent Guy:** writing – review and editing (equal). **Aurore Dougé:** supervision (equal), validation (lead), writing – review and editing (equal).

## 7 Conflicts of Interest

The authors have declared that no competing interests exist.

## Data Availability

Data sharing is not applicable to this article as no new data were created or analyzed in this study.
